# Interaction between *M. tuberculosis* Lineage and Human Genetic Variants Reveals Novel Pathway Associations with Severity of TB

**DOI:** 10.3390/pathogens10111487

**Published:** 2021-11-15

**Authors:** Michael L. McHenry, Eddie M. Wampande, Moses L. Joloba, LaShaunda L. Malone, Harriet Mayanja-Kizza, William S. Bush, W. Henry Boom, Scott M. Williams, Catherine M. Stein

**Affiliations:** 1 Department of Population and Quantitative Health Sciences, Case Western Reserve University, Cleveland, OH 44016, USA; mlm261@case.edu (M.L.M.); wsb36@case.edu (W.S.B.); smw154@case.edu (S.M.W.); 2 Department of Medical Microbiology, College of Health Sciences, Makerere University, Kampala, Uganda; eddie.wampande@mak.ac.ug (E.M.W.); mlj10@case.edu (M.L.J.); 3 Division of Infectious Diseases and HIV Medicine, Department of Medicine, Case Western Reserve University, Cleveland, OH 44106, USA; llm19@case.edu (L.L.M.); whb@case.edu (W.H.B.); 4 Department of Medicine and Mulago Hospital, School of Medicine, Makerere University, Kampala, Uganda; harriet.mayanja@mak.ac.ug

**Keywords:** tuberculosis severity, *M. tuberculosis*, population genetics, lineage-based GWAS, *M. tuberculosis*–human coevolution

## Abstract

Tuberculosis (TB) remains a major public health threat globally, especially in sub-Saharan Africa. Both human and *Mycobacterium tuberculosis* (MTBC) genetic variation affect TB outcomes, but few studies have examined if and how the two genomes interact to affect disease. We hypothesize that long-term coexistence between human genomes and MTBC lineages modulates disease to affect its severity. We examined this hypothesis in our TB household contact study in Kampala, Uganda, in which we identified three MTBC lineages, of which one, L4.6-Uganda, is clearly derived and hence recent. We quantified TB severity using the Bandim TBscore and examined the interaction between MTBC lineage and human single-nucleotide polymorphisms (SNPs) genome-wide, in two independent cohorts of TB cases (*n* = 149 and *n* = 127). We found a significant interaction between an SNP in *PPIAP2* and the Uganda lineage (combined *p* = 4 × 10^−8^). *PPIAP2* is a pseudogene that is highly expressed in immune cells. Pathway and eQTL analyses indicated potential roles between coevolving SNPs and cellular replication and metabolism as well as platelet aggregation and coagulation. This finding provides further evidence that host–pathogen interactions affect clinical presentation differently than host and pathogen genetic variation independently, and that human–MTBC coevolution is likely to explain patterns of disease severity.

## 1. Introduction

Pulmonary tuberculosis (TB) creates a large global public health burden, with 10 million active TB cases and 1.5 million deaths in 2020 [1]. Prior to the COVID-19 pandemic, *Mycobacterium tuberculosis* (MTB) was the mostly deadly pathogen on earth and has been for centuries [2]. While the global incidence of TB has generally trended downwards, TB continues to be a major driver of infectious disease mortality, and is a re-emerging infectious disease in Southeast Asia and sub-Saharan Africa [2]. Almost half (44%) of TB cases worldwide occur in sub-Saharan Africa. In Uganda alone, the location of our study, the incidence has been increasing since 2015, with almost 25,000 deaths per year due to TB [2]. TB is also the primary cause of death in human immunodeficiency virus (HIV)-positive people [3]. The bacterium, *Mycobacterium tuberculosis* (MTB)*,* which is transmitted via airborne droplets, causes most TB. Despite these troubling numbers, TB morbidity and mortality are not as injurious as they could be, as one-fourth to one-third of the entire globe is thought to be latently (asymptomatically) infected but only a small fraction of this number presents with disease.

The disease-causing *Mycobacterium* *tuberculosis* complex (MTBC) is organized into eight major lineages with distinct geographical distributions and timelines of human exposure [4,5]. Some lineages are ancestral (L1, L5, L6, L7, L8) while others are derived (L2, L3, and L4), and it has been observed that ancient lineages are less virulent than the modern ones. In addition to the major lineages, there are numerous sub-lineages that are thought to be recently diverged. The diversity of lineages and their historical coexistence with humans have led to the hypothesis that disrupted coevolution between the host and MTBC genes increases virulence [6,7,8,9,10,11].

Many of the derived lineages are thought to be descended from the MTBC L4 lineage, which is the most dispersed globally [4]. For example, at least 10 distinct sub-lineages of MTBC L4 arose in limited geographic areas. Relevant to our study, there is a sub-lineage found solely in Uganda and surrounding countries referred to as the MTBC L4.6/Uganda. Prior work showed no association between this MTB sub-lineage and the severity of disease, as determined by cavitary disease and extent of lung involvement [12,13]. These results present a major issue in assessing TB risk, namely, the extent to which pathogenicity or virulence is affected by the host, MTB, or both.

Given that there are ancient lineages that have co-existed with specific human populations for extended periods of time as well as novel lineages that arose recently, it was of particular interest how interactions between the two genomes impact severity. We and others have previously hypothesized that the severity of TB is affected by human–MTB coevolution that can be detected by examining specific interactions between human genes and MTB genes [10,11,14,15]. Studies examining human–MTB interactions at the population level are rare, and only studies of TB cases can determine host–MTB genome interactions as it impossible to know what MTB lineages an individual was exposed to prior to enrollment in a study if he/she does not have active disease. Additionally, most studies that have tried to contextualize the role of the two genomes with respect to each other in TB have examined MTB lineage and host genotypes as independent factors, usually without explicit interactions [16,17,18,19].

Under prudent exploitation and disrupted coevolution, long-term coexistence between the human genome and an *M. tuberculosis* complex (MTBC) lineage should result in decreased disease severity, whereas newly evolved or introduced strains should cause more severe disease [11,20,21,22]. Coevolution could drive concordant genetic variation of the two due to long-term coexistence and subsequent selection that promotes mutual adaptation. For example, it has been hypothesized that coevolution could result in mild disease or latent infection [21,23]. The hypothesis of coevolution between humans and MTBC has rarely been studied at the population level, but has been posited to be an important area of research [6,7,11,24,25,26,27,28,29]. The few studies that tried to address possible effects of coevolution are consistent with this hypothesis, with some MTB lineages appearing to be adapted to specific human populations [27,28]. 

There are other models and expected outcomes of coevolutionary theory, but we think that prudent exploitation is the most likely scenario for the case of coevolution between humans and MTB because: (1) most hosts who are infected do not develop active disease; (2) even fewer die from TB; and (3) the vast majority of TB infections worldwide are latent. With prudent exploitation, a newly derived MTB lineage that did not historically coexist with the human population in question should be associated with more severe disease [22]. Consistent with this possibility and the conditions that could lead to coevolution, humans and MTB have a very long history (between 6 and 80 thousand years) and most exposed people do not develop active disease (90–95%); hence, MTB appears to be a prudent exploiter [30]. However, few studies have identified clear evidence for coevolution at the population level [8,15,16,17,31,32,33].

Consistent with the prudent exploitation model of MTB infection, we recently showed that interaction between host variants in *SLC11A1* and MTB lineage was associated with disease severity [26]. Specifically, the derived Ugandan L4 sub-lineage (L4.6) caused more severe disease, but only in individuals with an ancestral *SLC11A1* genotype. This finding was notable in that *SLC11A1*, a well-studied candidate gene for TB susceptibility, was previously associated with TB in some but not all studies. Therefore, failure to replicate may be a function of human–MTB coevolution. While our earlier study focused on host candidate genes, here we assess this genome-wide. 

The focus of the present study is to assess possible coevolution via population genetic analyses under the model where the outcome (fitness) depends on the interaction of genetic variants between the species [34]. We measured TB severity using the TBscore that incorporates clinical severity using symptoms and clinical examination [35]. In our study, this outcome correlates with survival and therefore reproductive fitness, and it should be possible to measure disease severity as a product of human–MTB coevolution that has resulted from historical coexistence and demonstrated interaction of the two genomes [28,34]. Regression models were used to test for interaction (i.e., non-additive effect modification) between the genetic variation of the host and MTB lineage [11,25]. If present, statistical interaction between the mycobacterial lineage and human genotype can support the theory of prudent exploitation/coevolution if infection with an ancient lineage leads to less virulent disease in hosts carrying ancestral alleles [21,23]. Host–pathogen coevolution has been demonstrated in other organisms, such as *Helicobacter pylori,* using this approach [11,25].

Our primary hypothesis was that coevolution between human genotypes and pathogen lineage occurred and can be detected as a significant statistical interaction between the host variants and MTB lineage. Further, we postulated that more ancient MTB lineages will present with reduced severity in the presence of ancestral host alleles, and that the derived lineages should result in more severe disease, especially in the presence of the ancient host alleles. Our approach helped elucidate the degree to which the effects of lineage on severity are modified by and thus dependent on the genotype of the human host. 

## 2. Results

**Figure 1:**
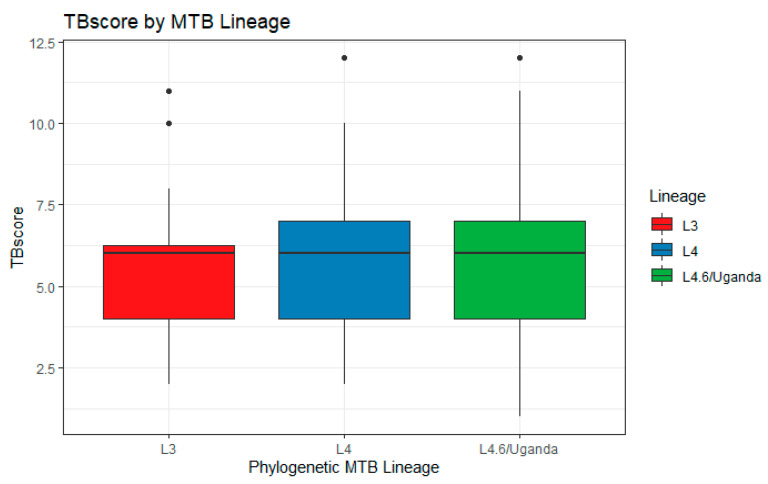
Boxplot of Severity by Lineage. Box plot shows the TBscore values for subjects who are infected with each of the 3 lineages present in our combined cohort. The midline of the box is the median. The box represents the inter-quartile range (25th to 75th percentiles) of the TBscore and the dots represent outliers.

The final study population included 276 subjects with data for human genotype, MTBC lineage, and the covariates of interest. We identified statistically significant differences between the two cohorts in the percentage of people who were HIV+ and the mean TBscore (Table 1). Males made up a larger proportion of subjects than females in the sample (which is consistent with prior studies showing that active TB is more prevalent among males), and most subjects were HIV-negative. All the models were adjusted for HIV status and sex. The distribution of MTB lineages showed no differences between cohorts, based on an ANOVA test (*p* = 0.66). The L4.6/Uganda lineage was the most prevalent in both cohorts (Table 1). The analysis of how lineage affects severity when considered singly showed that there was no association between lineage and severity (*p* = 0.65), and the boxplot confirms this graphically (Figure 1).

**Figure 2:**
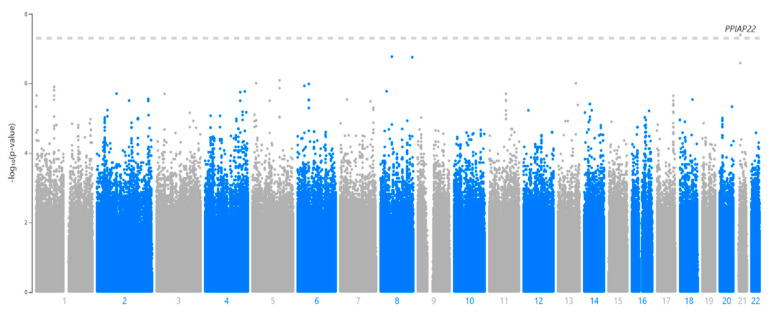
Manhattan Plot for Meta-Analytic *p*-values of Interaction Between SNP and L4.6/Ugandan Lineage. The Manhattan plot shows the inverse log(10) of the *p*-values for the association between interaction of each SNP and the L4.6/Ugandan lineage and TBscore on the *y*-axis, and the *x*-axis represents the physical location of each SNP on the chromosomes, which are in order from 1–22.

**Figure 3:**
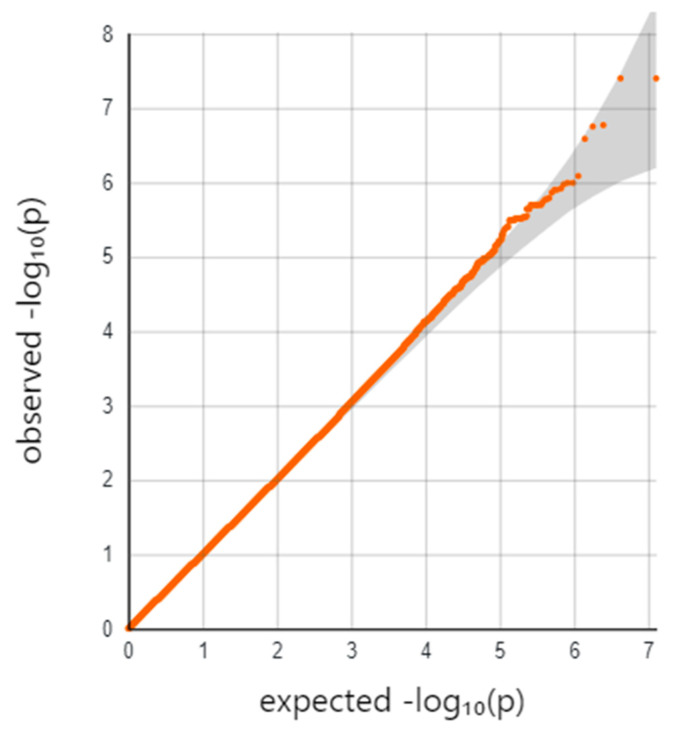
Q–Q Plot for Meta-Analytic *p*-Values for Interaction Between SNP and L4.6 Sub-Lineage. The quantile–quantile (Q–Q) plot shows the inverse log(10) of the observed *p*-values on the *y*-axis relative to what is expected if there was no association on the *x*-axis. Deviations above the line indicate an association with the outcome. If the line deviates at the low quantiles, then this is considered evidence to suggest genome-wide inflation of the test statistics, which typically indicates unmeasured confounding.

The analyses performed separately in each cohort did not show any significant interactions based on the GWAS threshold (Supplementary Materials Figures S1 and S2). Neither analysis showed any evidence of genome-wide inflation based on their Q–Q plots or genomic inflation parameters (λ < 1.0 for both) (Supplementary Materials Figures S3 and S4). The meta-analytic summary statistics also showed no signs of inflation in either the Q–Q plot nor the genomic control parameter, λ = 0.989, indicating little genome-wide inflation of the test statistics (Figure 2 and Figure 3).

**Figure 4:**
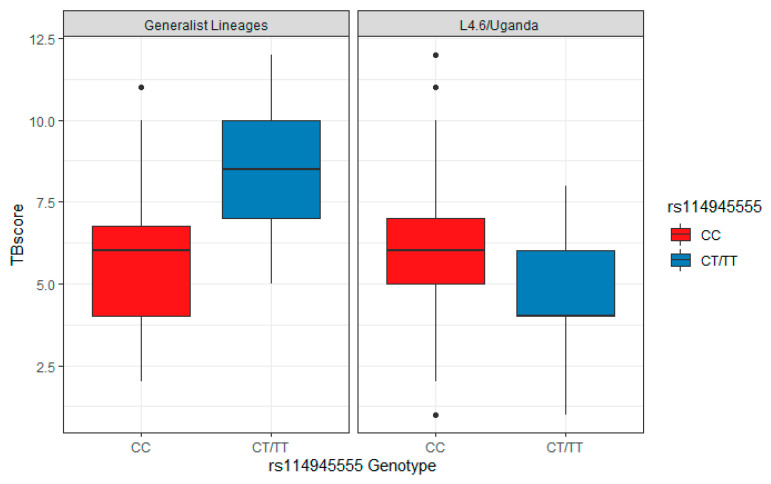
Interaction between rs114945555 and L4.6/Ugandan Lineage. There were also 6 SNP by lineage interactions with *p* < 1 × 10^−6^ (Table 4). Though none of these were below the GWAS threshold for significance, all were nominally significant (*p* < 0.05) and had consistent directions of effect (i.e., the beta value had the same sign) in both cohorts.

**Figure 5:**
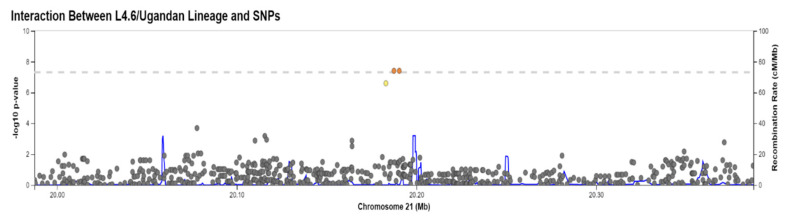
LocusZoom Plot for Region Surrounding rs114945555. The LocusZoom plot shows the region surrounding rs1848553 on chromosome 5, using an LD panel and reference genome from the AFR super-population in the 1000 G project. Yellow and orange indicate higher levels of LD.

In the meta-analysis, one SNP by lineage interaction met statistical significance at the GWAS threshold, rs114945555, an SNP on chromosome 21 that maps to *PPIAP22* (Beta = −4.13; *p* = 4.01 × 10^−8^) (Table 2, Figure 2). As this SNP did not show association with TBscore in the absence of an interaction term and the lineages did not show any association with TBscore, this result provided evidence that the interaction is driving the effect we see rather than either the first order SNP or lineage effects. This is also evident in Table 3, which shows that the strongest and most significant effects in the regression equations (which also includes the first-order effects of SNP and lineage) are the interaction terms. This most significant SNP (rs114945555) was not significant in the model without the interaction term (*p* = 0.28). The interaction term and the analysis were operationalized such that the L4.6/Ugandan lineage and the derived allele were both coded as 1, while the other lineages and ancestral alleles were coded as 0. Thus, the interaction term indicated that in the simultaneous presence of both the derived allele and the L4.6/Ugandan lineage, there is a 4.1-point decrease on the TBscore when the derived allele is present along with the derived MTB lineage relative to the derived allele in the presence of the generalist MTB lineages (Figure 4). This combination of lineage and genotype was observed in 25 of our subjects in this analysis. This is greater than the number of subjects (*n* = 12) who have the same genotype and the generalist lineages, but still represents a relatively small subset of our overall sample, as the derived allele has a relatively low frequency overall. There was an additional SNP on chromosome 21 (rs113863482) in near complete linkage disequilibrium (LD) (with completely identical beta and *p*-values) with rs114945555 that was also GWAS-significant. A third SNP in LD (rs112560854) was just below the GWAS threshold (*p* = 2.583 × 10^−7^) (Figure 5).

**Figure 6:**
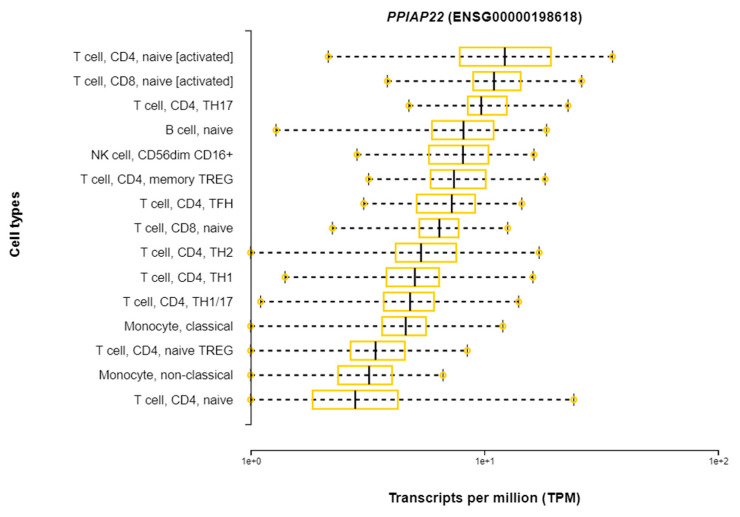
*PPIAP22* Expression in Immune Cells from DICE Database of Cell-specific Gene Expression.

**Figure 7:**
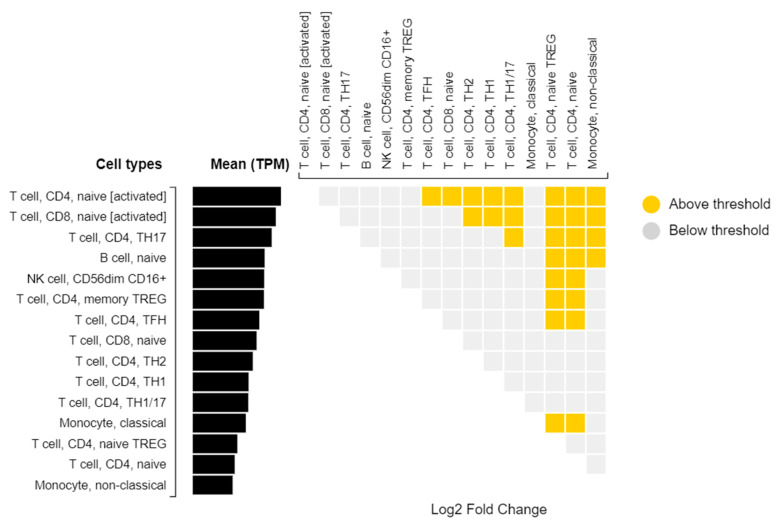
Differential Expression of PPIAP22 in Immune Cells from DICE Database of Cell-specific Gene Expression.

*PPIAP22* is a pseudogene and thus is not translated into a functional protein. It is a pseudogene for Cyclophilin A, a protein that is an important mediator of the inflammation response [36]. While *PPIAP22* is not translated into protein, it is transcribed into RNA, and evidence from the DICE database shows that it is expressed in a number of immune cells, with CD4+ T cells showing the highest expression (Figure 6 and Figure 7) [37].

The minor allele for rs114945555 exists almost exclusively in East Africa, based on the 1000 G project’s data, with a 7% MAF in the LWK population (a population based in Kenya). Outside of Africa, the MAF is 1% or 0% in every population and the LWK have the highest MAF in any African sub-population. In our data, the MAF was 9%, and this did not differ substantially with the East African data from 1000 G. The direction of the interaction in the L4.6/Ugandan lineage is consistent with the hypothesis concerning effect modification of the relationship between genotype and severity by MTB lineage. Specifically, in the presence of the ancestral generalist MTB lineage (i.e., L4) with which the population has had a longer exposure history, we hypothesized that the derived allele associates with more severe disease relative to the ancestral allele. In the presence of the more newly emergent specialist MTB lineage (i.e., L4.6/Uganda), we expected that the derived allele would associate with less severe disease relative to the ancestral allele. This was true for the interaction between rs114945555 (the only SNP showing a GWAS-significant interaction) and MTB lineage (Figure 4). 

Although we adjusted for HIV status in our analyses, we recognize that it is possible that these adjustments may not be sufficient. Therefore, we performed sensitivity analyses with only the HIV- subset to assess whether HIV status affected our results significantly. The results of our sensitivity analysis showed that our results between the analyses above with all samples and in only HIV- subjects were similar. While our *p*-values were slightly higher, this was likely due to the reduced sample size as an impact of excluding HIV+ patients. The lack of an effect of HIV status was further supported by the fact that the direction of effect was identical in the meta-analytic results for all SNPs in the results above. Further, the beta values were almost identical in analyses with all subjects and only those who are HIV- and the *p*-values were still significant in each cohort individually, despite not reaching the GWAS threshold after the exclusion of HIV+ subjects (Supplementary Materials Table S2). To further assess this point, we also conducted an ANOVA analysis to determine whether TBscore differed by HIV status, and it did not (*p* = 0.69). This lends additional weight to the argument that our inclusion of HIV+ subjects did not alter our major findings.

The MAGMA gene-based analysis included a total of 19,229 protein-coding genes represented by the SNPs in our data, and thus the threshold for significance was 2.6 × 10^−6^. Our results did not show any GWAS-significant effects for gene-level analysis, gene set enrichment, or tissue specificity, but the gene with the strongest interactions was *CA12* (*p* = 6.7 × 10^−3^), a carbonic anhydrase gene. The GSEA from the GENE2FUNC analyses showed significant enrichment for two of the chemical and genetic perturbation pathway gene sets (MsigDB c2) and two gene sets reported in the GWAS catalog (Table 5). Specifically, a breast cancer gene set containing “genes within amplicon 16p13 identified in a study of 191 breast tumor samples” and a gene set containing “Genes with copy number gains in primary neuroblastoma tumors” were significantly enriched in MsigDB c2. In the GWAS catalog, the nephrolithiasis and urolithiasis gene sets were significantly enriched.

**Figure 8:**
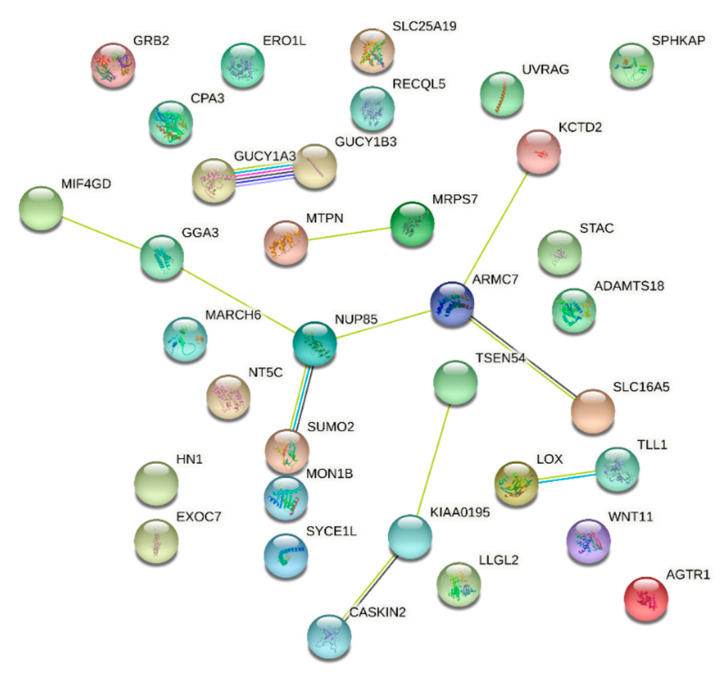
STRING Network for PPIs of eQTL Response Genes. This figure is a STRING diagram showing protein–protein interactions among the genes identified in my analysis. Lines represent an experimentally determined protein–protein interaction and multiple lines between the same two proteins indicate multiple interactions that have been identified, but multiple interactions between the same two proteins are still considered to be one edge.

For the SNPs showing interaction with the L4.6 sub-lineage (*p* < 1 × 10^−5^), we found that 21 of the 81 SNPs were eQTLs spanning 36 different genes for a total of 528 eQTL effects across the different tissues and databases (Figure 8, Supplementary Materials Table S1). A STRING DB analysis for PPIs among the genes showed significant enrichment for protein–protein interactions (PPI *p*-value = 0.0089; 11 edges) and was significantly enriched in six KEGG pathways (Table 6). From the STRING diagram and the table of KEGG pathways showing significant enrichment, we can see that many of these results are driven by the same proteins downstream of the genes from the eQTL analysis. Specifically, *GUCY1B3* and *GUCY1A3* are in every single pathway that was enriched, and the diagram shows interaction between them. *AGTR1* is also present in 4/6 of these KEGG pathways. Several of the enriched pathways appear to be related to renal function but are also closely tied to the regulation of vascular tone, endothelial permeability, and platelet aggregation. It has previously been suggested that these processes play a role in the response to infection and/or in the infection-induced inflammatory response [38,39,40,41,42].

Our previously published analysis of how the interaction between the L4.6/Ugandan sub-lineage and host genotype affects severity identified rs17235409, an exonic SNP within *SLC11A1*, a gene that has been well-studied in the context of TB susceptibility. Cohort 2 is comprised of the same subjects utilized in our prior study, so we examined the interaction between rs17235409 and the L4.6/Ugandan sub-lineage in Cohort 1 only, as replication in a distinct cohort. We did not have data available for rs17235409, so we examined SNPs within +/- 5 kb of *SLC11A1* on chromosome 2. We identified two SNPs with a *p*-value below 0.05: rs13390257 and rs116577076. Both are located within introns of *DIRC3* (which stands for disrupted in renal carcinoma gene 3), a long non-coding RNA gene characterized by its role in renal carcinoma (data not shown).

## 3. Discussion

Overall, our results showed that there are interactions between the human genome and MTB phylogenetic lineage that are associated with TBscore (i.e., active TB severity). The strongest interaction showed the same directionality of effect modification that we hypothesized and have previously published [26]. In these data, the difference in TB score between the two MTB lineage categories among those with the derived allele is very large. The beta value for the interaction term is −4.13. A TBscore difference of this magnitude is very clinically meaningful and represents a substantial difference in disease experience, level of disability, and risk of mortality. As the correlation with eventual mortality is a big driver of selection, our finding adds to the plausibility of the argument that coevolution is at play.

The GWAS-significant SNP, rs114945555, shows the highest MAF in the 1000 G population (LWK) that is closest to Uganda in geographical proximity. The fact that the derived allele, which associates with relatively less severe disease in the presence of the more recently derived MTB lineage, is more prevalent in a TB endemic region where the L4.6/Ugandan lineage is found, and is consistent with positive selection for this variant only where the new lineage exists, supports the coevolutionary hypothesis. If there is an evolutionary advantage to this allele in this specific population, then it is logical that it would increase in frequency. *PPIAPP22* is a pseudogene, and thus it is difficult to discern its functional role in the immune response to active TB. However, the evidence from the DICE database shows that it is differentially expressed in immune cells that are relevant to TB. Further, studies have shown that pseudo-genes may play an important regulatory role in human genetic diseases, with the potential to regulate protein-coding genes [43]. Some pseudogenes show a tissue-specific pattern of activation, and some pseudogene transcripts can be processed into short interfering RNAs (siRNAs) capable of regulating protein-coding genes [43]. In cancer and pharmacology, pseudogenes have been shown to have an important regulatory role in pathogenesis and are even considered therapeutic targets [44]. Thus, while *PIAPP22* is unlikely to play a direct role in the immune response, it may have important regulatory functions relevant to its interaction with MTB lineage.

The gene set analyses were also difficult to interpret, as breast cancer, kidney cancer, and stones found in the kidneys and urinary tract bear little obvious relevance to tuberculosis. However, it is possible that the pleiotropic effects of the gene pathways have not yet been elucidated, and there may yet be functional relevance of these gene sets. Many genes associated with cancer phenotypes may have general functions in cellular replication and metabolism. Thus, while they were shown as being important in cancer, they might have functions that affect a variety of phenotypes. Cancer-related gene sets have previously been shown to be enriched in several non-cancer phenotypes, and in previous studies a lack of apparent connection to TB does not necessarily indicate that there is no biological function [45]. Rather, it will be important to determine to what extent these genes may have functions outside of known associations. For the gene sets related to kidney function and kidney stones, pleiotropic effects may also be possible. Kidney complications in pulmonary TB are not uncommon and pulmonary TB has systemic effects on numerous organs because of generalized inflammation. Further, the kidneys play an important role in regulating homeostasis, particularly with respect to blood pressure and fluid levels. These may act to affect TBscore-related phenotypes. As such, it is possible that these results might yield interesting connections to TB severity upon further examination.

A subset of the eQTL SNPs that showed interaction with lineage appears to collectively be involved in the biological processes that regulate coagulation and vascular tone. The enrichment for processes shown in Table 6 appears to be driven primarily by the same three genes, *GUCY1B3, GUCY1A3*, and *AGTR1*. Five of these processes (renin, the renin–angiotensin system, gap junctions, vascular smooth muscle contraction, and the cGMP pathway) are involved in the regulation of blood pressure and vascular tone. Under normal physiological conditions, these pathways are part of a system that regulates blood pressure in response to factors such as fluid levels, electrolyte concentrations, stress, and cardiovascular output [46]. The vasculature also plays an important role in the response to infection, making changes that allow immune effector cells to move to where they are needed [40,41]. Under conditions of acute stress, and particularly in the context of infection-induced inflammation, this system can be perturbed and lead to acute hypertension (increase in blood pressure) that can reduce blood flow to vital organs [47]. In some cases, such as a cytokine storm in the context of septic shock, there can also be damage or death caused by hypotension, i.e., a decrease in blood pressure [48]. More specifically, the renin–angiotensin system (RAS) has been implicated as an important driver of the inflammation response in studies of lung damage and pulmonary vascular disease, where the changes in vascular tone of the micro-vasculature within the lungs can dictate the extent of alveolar damage and potential for recovery [49].

The cGMP pathway is also important to platelet homeostasis and the coagulation cascade and has previously been shown to be an important regulator of the cell migration and T cell polarization in the host immune response [50,51]. Platelets play an important role in the immune and inflammatory response to infection [38,42]; the dysregulation of the system as a result of infection has been well-documented in other infectious diseases [39], and active TB patients experience a pro-coagulatory state as a result of systemic inflammation [52,53]. cGMP pathways have also been directly implicated as a driver of inflammation in the context of infectious disease, and specifically in the context of pulmonary infectious diseases. For example, they may represent a therapeutic target that can mitigate the most severe forms of COVID-19 [50,54,55]. Thus, the enrichment results indicate that the consequences of systemic inflammation may be driving more severe manifestations of TB disease. This is important, as prior studies of TB susceptibility have primarily identified immune response genes (though there is some overlap), indicating that different biological processes may be at play in susceptibility and severity.

This study was not without limitations. This sample was limited to 279 subjects across the two cohorts, which is relatively small compared to many modern GWAS studies, which can include thousands of subjects. While we were able to detect one GWAS-significant interaction, there were six other SNP by lineage interactions with *p* < 1 × 10^−6^ and consistency across our two cohorts. Thus, it is possible that with a larger sample size, these interactions might have achieved GWAS significance. Nonetheless, we were able to detect one interaction that may play a role in TB severity, and we generated a list of 91 SNPs with *p* < 1 × 10^−5^ that showed enrichment for multiple gene sets. Finally, we were able to provide further evidence for the phenomenon of human–MTB co-evolution and demonstrate how this can be performed in genetic epidemiology studies.

## 4. Materials and Methods

### 4.1. Study Participants

The data in this study were drawn from the Kawempe Community Health Study (KC Health Study) in Kampala, Uganda [54]. The KC Health study enrolled 3818 total participants that included 1045 active TB cases, from which the study population in this paper is derived. Our analyses included only individuals with available data on human genotype, clinical symptoms related to active TB severity, and information on the specific lineage/sub-lineage of MTBC with which the patient is infected. The TBscore was developed for adults and may not be appropriate for individuals under 15. Thus, our sample was limited to subjects 15 years old and older. We examined two samples collected separately as part of this study that will be referred to as Cohort 1 and Cohort 2 (*n* = 149 and *n* = 127, respectively). The two cohorts were recruited at different times and genotyping of the humans was performed using different arrays for them, as described below. Ascertainment criteria did not differ.

All TB cases were culture-confirmed positive based on the isolation of MTB in sputum or gastric samples, and the clinical characteristics were collected as part of the visit during which subjects were diagnosed with active TB. Chest radiographs (X-rays) were performed at the Uganda Cancer Institute on subjects with confirmed active TB. The study protocol was approved by the National HIV/AIDS Research Committee of Makerere University and the institutional review board at University Hospitals Cleveland Medical Center. Final clearance was given by the Uganda National Council for Science and Technology. All participants provided written informed consent. Additional details about the original study protocol are described elsewhere [54]. The two cohorts differed in percentage of HIV-positive individuals (Table 1); therefore, HIV status was used as a covariate in all regression models. Previous analyses of microsatellite data from these cohorts indicated no substantial population substructure, as previous principal component (PC) analyses have corroborated [26,55].

### 4.2. Human Genotyping and QC

Cohort 1 was genotyped on the Illumina Infinium Mega^EX^ chip, comprising 2.1 M markers genome-wide. For Cohort 2, we used the Illumina HumanOmni5 microarray comprising 4.3 M markers genome-wide, offering high genome-wide coverage of common genetic variation even within African populations [56]. The difference in choice of genotyping chips was based only on commercial availability at the time of genotyping. Only SNPs that had a call rate greater than 0.98, MAF > 0.05, and did not show deviation from the Hardy–Weinberg equilibrium (*p* < 10^−6^) prior and subsequent to imputation in both samples were used in the analysis. The genotype data for Cohort 1 initially included 2,036,060 SNPs. After QC and prior to imputation, there were 717,705 SNPs remaining. After imputation and QC, we had 8,146,092 SNPs. The genotype data for Cohort 2 included 2,989,642 SNPs to start. After QC, 1,931,961 SNPs remained prior to imputation. After imputation and another round of QC thresholds, there were 9,626,100 SNPs. These were the final numbers of SNPs in each cohort prior to meta-analysis. The total number of SNPs that overlapped between the two cohorts was 6,421,278. Principal components were computed using Plink v1.9 [57].

### 4.3. Determination of MTB Lineage

MTB was isolated from the sputum of each of these subjects, and lineages were classified according to lineage-identifying SNPs using real-time PCR and validated with long sequence polymorphism (LSP) PCR and sequencing [12,58]. Lineage was determined from three SNPs that accurately distinguish the MTBC L4.6 Uganda, MTBC L3, and MTBC L4 lineages, as previously described [4,6,7]. The classifications delineated by these SNPs were then compared to previously established LSP-based lineages to validate these distinctions. In the context of this study setting, the relevant MTBC lineages were MTBC Lineage 4 (referred to in this paper as L4/Non-Uganda), MTBC Lineage 3 (L3 also known as Central Asian Strain), and MTBC Lineage 4.6/Uganda, which is a sub-lineage of MTBC L4 that is only found in Uganda and the countries immediately surrounding it [4,6,7,9,12]. The MTBC L4.6/Uganda is the most recently evolved of the three, a sub-lineage of the MTBC L4 generalist lineage, and is unique to this part of Africa [4,9].

SNP and LSP-based phylogeny have been proven to be consistent in multiple studies of MTBC sub-lineages, and the body of literature on MTBC lineages indicates that this is an excellent method for differentiating the MTBC L4 sub-lineages [6,28,59]. Low sequence variation and the lack of horizontal transfer make SNPs and LSPs a method well-suited to distinguish lineages, and this approach has been previously validated and published [6,12,13]. MTBC L4.6 is the most common lineage among active TB cases in this cohort [6,7,9,12]. This sub-lineage has been shown to have highly conserved T-cell epitopes (i.e., a lower proportion of variable epitopes) and a much smaller geographic range than non-specialized lineages, indicating that it may be adapted to a specific host population(s) [4].

### 4.4. Statistical Analysis

To assess the association between variants and TBscore, we utilized a linear regression model with sex and HIV status as covariates in Plink v1.9 software. For the interaction term between lineage and human genotype, we chose to operationalize lineage as a binary variable. Each subject is coded as 1 for the L4.6/Ugandan lineage or as 0, which encompasses the L4/Non-Uganda and MTBC L3/Central Asian Strains together. As the L4/Non-Uganda and L3/Central Asian lineages have a longer history of human contact compared to the L4.6/Uganda, which is a newer sub-lineage, we can examine coevolution as we are contrasting a lineage that is more recent, relative to the two older lineages (MTBC L3 and MTBC L4). As we expected a longer historical coexistence to associate with lesser severity and the introduction of a newer sub-lineage to associate with greater severity, we grouped the two older lineages together. This also affords greater power to detect a difference than if we were to examine all 3 lineages independently. A power calculation showed that we have 70% power to detect a 2.5 point change in TBscore due to the interaction (the beta value from our previous paper) at *p* = 5 × 10^−6^. The regression equation for modeling co-evolution was: Y = β_0_ + β_1_X_1_ + β_2_X_2_ + β_3_X_3_ + β_4_X_4_ + β_5_ (X_2_X_3_) + ε, where X_1_ = HIV-positive status, X_2_ = L4.6/Uganda lineage, X_3_ = SNP, X_4_ = sex. In these analyses, we used a dominant model of inheritance (homozygous minor allele and heterozygotes as effect vs. the homozygous major allele as referent).

We then combined the summary statistics from the two cohorts to generate meta-analytic *p*-values. To determine meta-analytic *p*-values and beta coefficients across the two cohorts, we utilized random effects meta-analysis with inverse variance weighting. Based on the Cochrane handbook recommendations, all variants with an I^2^ > 40% were excluded from the analysis to reduce heterogeneity between the cohorts [60]. To be considered GWAS-significant, the interaction term between an SNP and the L4.6 sub-lineage had to have a *p* < 0.05 in both cohorts, the sign of the beta value had to be the same in both cohorts, and the meta-analytic *p*-value had to be below 5 × 10^−8^, the canonical GWAS threshold. To be included in further enrichment and annotation analyses, the meta-analytic *p*-value had to be below 1 × 10^−5^. We chose this threshold because previous studies have shown that some variants that do not meet the GWAS threshold may still have important regulatory or biological functions in some cases and may be worthy of further study and follow-up, especially in the context of gene regulation [59,61,62].

In order to determine the extent to which our results were sensitive to the inclusion of both HIV-positive and negative subjects, we ran the same analyses described above among solely the HIV- subjects and compared these meta-analytic summary statistics to those in our original analyses. While our original analysis already adjusted for HIV status as a covariate in the regression equations used to assess the interaction between the interaction of SNP variants and MTB sub-lineage and the association with TB severity, such an analysis may help show whether or not our results were affected by the inclusion of both HIV-negative and positive subjects.

We used FUMA GWAS to annotate and enrich our SNPs below the threshold. Analyses performed through FUMA included gene mapping, regulatory annotation, tissue specificity, MAGMA analysis (gene-based analysis), gene set enrichment, and pathway analyses [63]. In addition to FUMA, we utilized GeneCards, Ensembl, DICE, and STRING DB to help annotate and enrich our results with respect to function, expression, and downstream protein interactions [36,37].

## Figures and Tables

**Table 1:** Study Population.

	Cohort 1 *n* = 149	Cohort 2 *n* = 127	Total	*p*
Mean Age (SD)	28.7 (8.1)	28.7 (9.8)	28.7 (9.0)	0.99
# Male (%)	81 (54.4)	73 (57.5)	154 (55.8)	0.69
# HIV+ (%)	31 (24.4)	15 (10.1)	46 (16.7)	0.0025
Mean TBscore (SD)	6.2 (2.1)	5.4 (2.2)	5.8 (2.2)	0.0032
# L4.6/Ugandan (%)	93 (62.4)	75 (59.1)	168 (60.9)	0.66
# L4 (%)	15 (10.1)	17 (13.4)	32 (11.6)	
# L3 (%)	39 (26.2)	31 (24.4)	70 (25.3)	

Differences in age and TBscore were analyzed using a Student’s *t*-test and differences in the percentage of males and HIV+ subjects were analyzed using Z-statistics. Differences in the distribution of lineages were analyzed using a Chi-squared test. For all tests, *p* < 0.05 was considered a significant difference.

**Table 2:** GWAS-Significant Loci for Interaction between SNP and L4.6 Sub-Lineage.

SNP	CHR:BP	Ref/Alt	Gene *	Location	MAF (LWK)	*p*-Value
rs114945555	21:20187488	C/T	*PPIAP22*	Intergenic	7%	4.0 × 10^−8^

* rs114945555 mapped to *PPIAP22* in LocusZoom and FUMA GWAS web applications. Minor allele frequencies were ascertained from the 1000 G project using Ensembl v104.

**Table 3:** rs114945555 × Lineage Interaction across Cohorts 1 and 2.

	Cohort 1		Cohort 2		Combined	
	β	*p*	β	*p*	β	*p*
rs11945555 s119 (β_3_)	1.98 (0.28,3.68)	0.024	2.94 (1.27,4.61)	7 × 10^−4^	2.47	4.80 × 10^−5^
L.4.6/Ugandan (β_2_)	0.59 (−0.13,1.3)	0.11	0.57 (−0.24,1.38)	0.17	0.58	0.03
Sex (β_4_)	0.49 (−0.17,1.14)	0.15	1.09 (0.32,1.85)	0.0061	0.75	0.012
HIV+ status (β_1_)	0.60 (−0.48,1.69)	0.28	−0.14 (−1.02,0.74)	0.75	0.16	0.66
rs11945555 × L4.6/Ugandan (combination of CT/TT and L4.6 Ugandan) (β_5_) †	−3.77 (−5.79,−1.75)	3.62 × 10^−4^	−4.53 (−6.68,−2.38)	6.79 × 10^−5^	−4.13	4.00 × 10^−8^

Regression Model: Y = β_0_ + β_1_X_1_+ β_2_X_2_ + β_3_X_3_ + β_4_X_4_ + β_5_ (X_2_X_3_) + ε. X_1_ = HIV+ Status, X_2_ = L4.6/Ugandan lineage, X_3_ = rs114945555, X_4_ = Sex. † Model of inheritance (CT/TT vs. CC as referent).

**Table 4:** SNPs Above GWAS Threshold with *p* < 1 × 10^−6^ (Meta-analytic *p*-values and Betas).

Chr	Bp	SNP	*p*	β	Gene	Location
5	17775271	rs369093426	9.82 × 10^−7^	3.2095	*LOC105374666*	Intron
5	121258204	rs76190408	8.14 × 10^−7^	3.0447	None	Intergenic
8	50958714	rs203964	1.70 × 10^−7^	2.7085	*SNTG1*	Intron
8	141085471	rs56990580	1.77 × 10^−7^	−3.3244	*TRAPPC9*	Intron
13	985899842	rs8000063	9.82 × 10^−7^	2.6203	None	Intergenic
21	20182990	rs112560854	2.58 × 10^−7^	−3.7805	None	Intergenic

Chr–chromosome number, Bp = basepair location. Gene and location were ascertained using Ensembl v.104.

**Table 5:** Gene Set Enrichment for SNPs by Lineage Interactions.

GeneSet	*N*	*n*	*p*-Value	Adjusted *p*	Genes	Database
Breast Cancer Amplicon	329	24	3.5 × 10^−23^	1.15 × 10^−19^	*GPRC5C, TMEM104, GRIN2C, FADS6, CDR2L, KCTD2, ATP5H, SLC16A5, ARMC7, NT5C, HN1, SUMO2, NUP85, GGA3, MRPS7, MIF4GD, SLC25A19, GRB2, KIAA0195, CASKIN2, TSEN54, LLGL2, RECQL5, EXOC7*	Chemical and Genetic Perturbations (MsigDB c2)
Neuroblastoma Copy Number Up	181	9	8.92 × 10^−8^	0.000147	*GPRC5C, ATP5H, ARMC7, HN1, SUMO2, NUP85, MRPS7, SLC25A19, TSEN54*	
Nephrolithiasis	8	3	4.33 × 10^−6^	7.87 × 10^−3^	*INMT, FAM188B, AQP1, AQP1*	GWAS Catalog
Urolithiasis	17	3	5.12 × 10^−5^	0.046	*TFAP2B, INMT, FAM188B*	

*N* indicates the total number of genes in the set while *n* shows the number of genes that SNPs mapped to in our GWAS data. Gene sets were queried using FUMA GWAS. The adjusted *p* is an FDR-corrected *p*-value based on the number of gene sets examined.

**Table 6:** Significantly Enriched KEGG Pathways for eQTL Response Genes.

KEGG Pathway	# Genes	# In Set	Strength	FDR	Matching Proteins
Renin secretion	3	63	1.44	0.0166	*GUCY1B3, GUCY1A3, AGTR1*
Gap junction	3	87	1.3	0.0207	*GUCY1B3, GUCY1A3, GRB2*
Renin-angiotensin system	2	23	1.7	0.0234	*CPA3, AGTR1*
Vascular smooth muscle contraction	3	119	1.16	0.0251	*GUCY1B3, GUCY1A3, AGTR1*
cGMP-PKG signaling pathway	3	160	1.03	0.0459	*GUCY1B3, GUCY1A3, AGTR1*
Purine metabolism	3	173	1	0.0474	*NT5C, GUCY1B3, GUCY1A3*

## Data Availability

Because of the IRB restriction on the data from Uganda, individual level data are only available upon request from the Uganda Genetics of TB Data Access Committee by contacting Dr. Sudha Iyengar (ski@case.edu).

## Supplementary Materials

The following are available online at https://www.mdpi.com/article/10.3390/pathogens10111487/s1, Figure S1: Manhattan Plot for Interaction Between SNP and L4.6/Ugandan Lineage in Cohort 1, Figure S2: Manhattan Plot for Interaction Between SNP and L4.6/Ugandan Lineage in Cohort 2, Figure S3: Quantile–Quantile Plot for Interaction Between SNP and L4.6/Ugandan Lineage in Cohort 1, Figure S4: Quantile–Quantile Plot for Interaction Between SNP and L4.6/Ugandan Lineage in Cohort 2, Table S1: SNPs in eQTL analysis, Table S1. SNPs in eQTL analysis (in excel format) Table S2: Top SNPs from Original Analysis Compared to Summary Statistics from Sensitivity Analysis Including only HIV- Subjects.
