# Supplementary material for: Interaction between M. tuberculosis Lineage and Human Genetic Variants Reveals Novel Pathway Associations with Severity of TB
Source: Pathogens. 2021 Nov 15;10(11):1487. doi: 10.3390/pathogens10111487 (PMC8617877; doi:10.3390/pathogens10111487)
Supplement: Supplementary file 1 [file pathogens-10-01487-s001.zip › Supp Fig and Table S2.pdf]

Supplemental Figure S1. Manhattan Plot for Interaction Between SNP and L4.6/Ugandan Lineage in Cohort 1

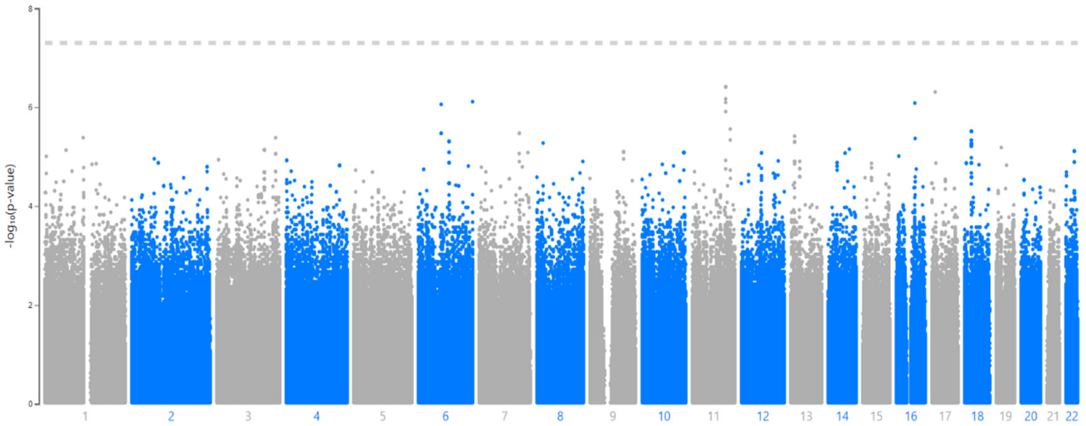

The Manhattan plot shows the inverse log(10) of the p-values for the association between interaction of each SNP and the L4.6/Ugandan lineage and TBscore on the y-axis and the x-axis represent the physical location of each SNP on the chromosomes, which are in order from 1-22.

Supplemental Figure S2. Manhattan Plot for Interaction Between SNP and L4.6/Ugandan Lineage in Cohort 2

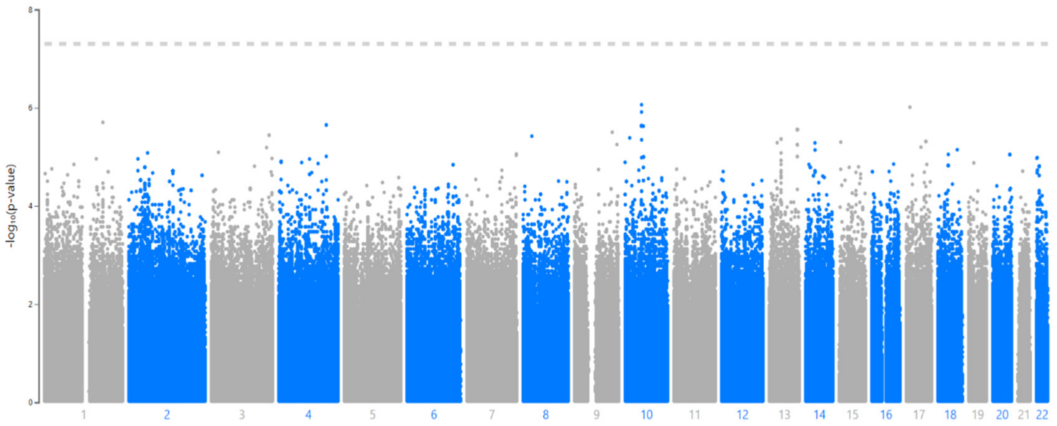

The Manhattan plot shows the inverse log(10) of the p-values for the association between interaction of each SNP and the L4.6/Ugandan lineage and TBscore on the y-axis and the x-axis represent the physical location of each SNP on the chromosomes, which are in order from 1-22.

Supplemental Figure S3. Quantile-Quantile Plot for Interaction Between SNP and L4.6/Ugandan Lineage in Cohort 1

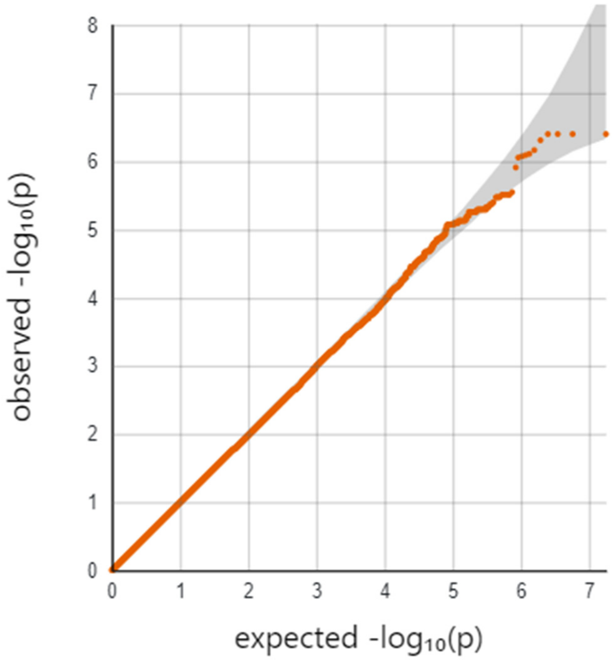

The quantile-quantile (Q-Q) plot shows the inverse log(10) of the observed p-values on the Y-axis relative to what is expected if there was no association on the x-axis. Deviations above the line indicate an association with the outcome. If the line deviates at the low quantiles, then this is considered evidence to suggest genome-wide inflation of the test statistics, which typically indicates unmeasured confounding.

Supplemental Figure S4. Quantile-Quantile Plot for Interaction Between SNP and L4.6/Ugandan Lineage in Cohort 2

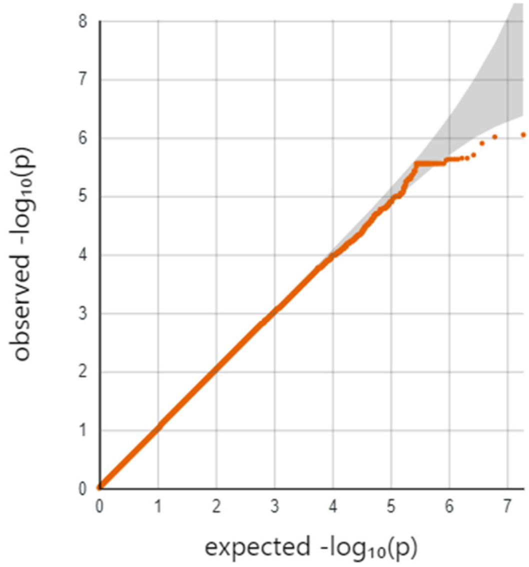

The quantile-quantile (Q-Q) plot shows the inverse log(10) of the observed p-values on the Y-axis relative to what is expected if there was no association on the x-axis. Deviations above the line indicate an association with the

outcome. If the line deviates at the low quantiles, then this is considered evidence to suggest genome-wide inflation of the test statistics, which typically indicates unmeasured confounding.

Supplemental Table S2. Top SNPs from Original Analysis Compared to Summary Statistics From Sensitivity Analysis Including only HIV- Subjects

CHR	BP	SNP	P (HIV-)	β (HIV-)	P (All)	β (All)
21	20187488	rs114945555	3.13E-07	-4.16	4.00E-08	-4.13
5	17775271	rs369093426	1.20E-06	3.34	9.82E-07	3.21
5	121258204	rs761904408	7.61E-06	3.15	8.14E-07	3.04
8	50958714	rs203964	3.31E-06	2.73	1.70E-07	2.71
8	141085471	rs56990580	3.35E-06	-3.28	1.77E-07	-3.32
13	98589842	rs8000063	1.23E-05	2.66	9.82E-07	2.62
21	20182990	rs112560854	2.03E-06	-3.76	2.58E-07	-3.78
